# Association between cataract and genetic polymorphisms of *GSTM1*, *GSTT1*, and *GSTO2* with respect of work place

**Published:** 2012-07-18

**Authors:** Iraj Saadat, Zainab Ahmadi, Majid Farvardin-Jahromi, Mostafa Saadat

**Affiliations:** 1 Department of Biology, College of Sciences, Shiraz University, Shiraz, Iran; 2 Institute of Biotechnology, Shiraz University, Shiraz, Iran; 3 Poostchi Eye Research Center, Department of Ophthalmology, Shiraz University of Medical Sciences, Shiraz, Iran

## Abstract

**Purpose:**

To investigate whether genetic polymorphisms of glutathione S-transferases (*GSTM1*, *GSTT1*, and *GSTO2*) in relation to the work place contribute to the development of cataract.

**Methods:**

The present case-control study consisted of 186 patients (108 females, 78 males) with cataract and 195 gender-matched healthy controls (111 females, 84 males) were randomly selected from unrelated volunteers in the same clinic. The *GSTM1*, *GSTT1*, and *GSTO2* genotypes were determined using polymerase chain reaction (PCR) based methods.

**Results:**

The null genotype of *GSTM1* increased the risk of cataract (OR=1.51, 95%CI: 1.01–2.26, p=0.045). The prevalence of *GSTT1* and *GSTO2* genotypes was similar between cases and controls. There was significant difference between cases and controls for work place (χ^2^=4.16, df=1, p=0.041). Genetic polymorphisms (*GSTM1*, *GSTO2*) and work place that were significant by p<0.3 in the univariate analysis were included in the analysis for investigating the additive effects of the genotypes and work place on risk of cataract. Statistical analysis showed that the risk of cataract increased as a function of number of putative high risk factors (χ^2^=8.001, p=0.005).

**Conclusions:**

This finding suggests that the polymorphisms of *GSTM1* and *GSTO2* and also work place may act additively for developing cataract.

## Introduction

Cataract is a multifactorial disease which is associated with many environmental [1] and genetic variations [2-4]. Epidemiologic studies have shown that cataract is associated with environmental factors such as exposure to sunlight and ultraviolet B light [1]. Oxidative stress as a result of increased generation of reactive oxygen species and free radicals in the lens has been considered one of the main causes of senile cataract [5,6]. The toxic effects of oxidative stress during cataractogenesis can be alleviated by cellular defense mechanisms. Glutathione S-transferases (GSTs; EC 2.5.1.18) are a superfamily of ubiquitous multifunctional cytosolic soluble detoxification enzymes that can catalyze the conjugation of reduced glutathione to various xenobiotics and endobiotics [7-9]. GSTs play important roles in cellular protection against oxidative stress. Human GSTs divided into different classes; including mu, theta and omega. Genetic polymorphisms in genes encoding *GSTM1* (a member of class mu; OMIM 138350), *GSTT1* (a member of class theta; OMIM 600436), and *GSTO2* (a member of class omega, OMIM: 612314) have been defined [7-9]. The *GSTM1–0* (*GSTM1* null) and *GSTT1–0* (*GSTT1* null) alleles represent deletions of *GSTM1* and *GSTT1* genes, respectively. The functional consequences of homozygosity for the *GSTM1* and *GSTT1* null alleles (null genotypes) are obvious in terms of enzyme activity: no gene, no enzyme, and no activity [7,8]. The homozygotes and heterozygotes for the wild type allele named positive genotypes. Association between polymorphisms of several classes of glutathione S-transferases (GSTs such as *GSTM1*, *GSTT1*) and risk of cataract have been studied [10-12]. Based on a meta-analysis it is suggested that *GSTM1* and *GSTT1* null genotypes are both associated with increased risk for senile cataract in Asian populations but not in Caucasian populations [12].

The *GSTO2* have poor activity with common GST substrates, but exhibit novel glutathione-dependent thioltransferase, dehydroascorbate reductase, and monomethylarsonate reductase activities [9]. In human, the *GSTO2* is polymorphic with an N142D substitution in the coding region [9]. It is reported that the GSTO2 142Asp (142D) variant allozyme showed 20% reduction in level of expression compared with the level of the GSTO2 wild type (142N) allozyme [13]. It is shown that As_2_O_3_ inhibits the growth and induces apoptosis of cultured human lens epithelium cells [14]. There was an increasing prevalence of cataract with the increase in exposure to ingested arsenic [15]. Taken together, it may conclude that the *GSTO2 N142D* polymorphism may have an effect on individual susceptibility to many multifactorial diseases, such as cataract.

There is no study investigating the additive effects of these polymorphisms in relation to work place (as an indicator for exposure to sunlight) and the cataract risk. To get more insight into the possibility of contribution of these genetic variations to susceptibility of senile cataract, the present study was performed.

## Methods

### Subjects

Patients with senile cataract were recruited from Khalili Hospital Ophthalmic Clinic in Shiraz, Iran. All 186 subjects with cataract had severe visual disturbance and their corrected visual acuities were under 0.1. We excluded patients with secondary cataract due to diabetes, trauma, steroid administration, and other causes. The gender-matched control subjects were collected from unrelated volunteers in the same clinic. Iranian population is one of the most heterogeneous populations [16,17]. Therefore, we selected our patients and controls from the same ethnical religious group (Persian Muslims living in Fars province, southern Iran). Both groups had no history for cancer and asthma. The mean age of the cataract patients and the controls was 67.2±9.7 years and 58.1±8.8 years, respectively. There was significant difference between cases and controls for age distribution of participants (t=9.54, df=379, p<0.001).

Informed consent was obtained from each subject before the study. This study was approved by the local ethics committee. Informed consent was obtained from all participants.

The study subjects were divided into two groups: outdoor (farmers, drivers, etc) and indoor (housewives, teachers, etc) according to their job titles. The outdoor and indoor patients were occupationally exposed to sunlight and not exposed to sunlight, respectively.

### Extraction of DNA and genotyping analysis

Blood samples were obtained from patient and control groups. Immediately after collection, whole blood was stored at −20 °C until use. Immediately after blood collection, whole blood was stored at −20 °C until use. Genomic DNA for PCR was isolated from whole blood using the thawed blood samples [17]. The primers and PCR conditions for determining *GSTM1*, *GSTT1* and *GSTO2* genotypes were the same as those reported previously [18] and shown in Table 1. To ensure laboratory quality control, two independent readers interpreted the gel photographs. Any sample with ambiguous results (generally due to low PCR yield) was re-tested, and a random selection of 15% of all samples was repeated. No discrepancies were discovered upon replicate testing.

**Table 1:** Primers used for genotyping.

**Primer name**	**Primer sequence**	**Melting temperature (°C)**	**Product size (bp)**
GSTM1 (F)	5'-TTCCTTACTGGTCCTCACATCTC-3'	65.5	219
GSTM1 (R)	5'-TCACCGGATCATGGCCAGCA-3'		
GSTT1 (F)	5'-GAACTCCCTGAAAAGCTAAAGC-3'	65.5	459
GSTT1 (R)	5'-GTTGGGCTCAAATATACGGTGG-3'		
b-globin (F)	5'-CAACTTCATCCACGTTCACC-3'	65.5	268
b-globin (R)	5'-GAAGAGCCAAGGACAGGTAC-3'		
GSTO2 (F)	5'-AGGCAGAACAGGAACTGGAA3'	65.0	185
GSTO2 (R)	5'-GAGGGACCCC TTTTT GTACC-3'		

### Statistical analysis

A χ^2^ test was performed for *GSTO2* polymorphism to determine if the control samples demonstrated Hardy–Weinberg equilibrium. Unconditional logistic regression was used to calculate ORs and 95% CI for cataract risk associated with the genetic polymorphisms of *GSTs*. Considering the significant age difference between patients and controls, in further analysis, logistic regression was used to calculate ORs and 95% CI for the various genotypes after adjusting for age. In these analyses the reference group consisted of putative low risk genotypes (positive genotype of *GSTM1*, positive genotype of *GSTT1*, and NN genotypes of *GSTO2*).

Data on work place in the control subjects were missed for some participants. To study the potential effect of the work place on cataract risk when investigated the risk associated with genotypes of *GSTs* polymorphisms, the “sensitivity analysis” was used [19]. For doing “sensitivity analysis” we test two assumptions. First, all missing data of work place in control group were assumed outdoor. Second, 50% of missing data for work place in control group were assumed indoor.

Statistical analysis was performed using the Statistical Package for Social Sciences (version 11.5; SPSS Inc., Chicago, IL). In multiple comparisons, the error rate is often much larger than the error rate applied to each single analysis. This can result in the declaration of spurious effects as significant. Bonferroni adjustment, requires that each analysis be performed using an α/*k* Type I error rate, where *α=*0.05 and *k* is the number of comparisons made (here *k*=3; α/*k*=0.017). However, this results in a very conservative estimate of the statistical significance of each evaluation.

## Results and Discussion

Table 1 shows the genotype distribution of the *GSTO2* N142D, *GSTM1*, and *GSTT1* genetic polymorphisms in cataract patients and healthy controls. Control subjects for the genotypic frequencies of the *GSTO2* polymorphism were in Hardy–Weinberg equilibrium (χ^2^=0.103, df=1, p=0.748). Logistic regression analysis showed that there was no significant association between *GSTO2* polymorphism and susceptibility to cataract (Table 2). Frequency of the *GSTM1* null genotype among cataract patients and control subjects were 55.9 and 45.6%, respectively. The null genotype of *GSTM1* increased the risk of cataract (OR=1.51, 95%CI: 1.01–2.26, p=0.045). The prevalence of *GSTT1* null genotype was similar between cases and controls (26.3 versus 29.2%; OR=0.86, 95% CI: 0.53–1.35, p=0.952; Table 2).

**Table 2:** Association between *GSTO2* (N142D), *GSTM1*, and *GSTT1* polymorphisms and cataract risk.

**Polymorphisms**	**Control**	**Case**	**OR**	**95%CI**	**p-value**	Adj. OR*	**95%CI**	**p-value**
**N142D *GSTO2* polymorphism**								
NN	87	73	1.0	Reference	-	1.0	Reference	-
ND	85	83	1.16	0.75–1.79	0.493	1.19	0.73–1.94	0.472
DD	23	30	1.55	0.83–2.90	0.167	1.91	0.96–3.80	0.065
ND+DD	108	113	1.24	0.82–1.87	0.289	1.34	0.85–2.12	0.204
***GSTM1* polymorphism**								
Positive	106	82	1.0	Reference	-	1.0	Reference	-
Null	89	104	1.51	1.01–2.26	0.045	1.61	1.02–2.52	0.039
***GSTT1* polymorphism**								
Positive	138	137	1.0	Reference	-	1.0	Reference	-
Null	57	49	0.86	0.53–1.35	0.952	0.93	0.56–1.54	0.794

* Note: Adjusted OR for age of participants.

Patients and control subjects worked in outdoor, 25.8 and 16.7%, respectively. Work place significantly differed between cases and controls (χ^2^=4.16, df=1, p=0.041). It should be mentioned that work place was missing for 34 participants of controls.

Genetic polymorphisms (*GSTM1*, *GSTO2*) and work place that were significant by p<0.2 in the univariate analysis were included in the analysis for investigating the additive effects of the genotypes and work place. Table 3 showed the profiles of GSTs genotypes in control and cataract patient groups with respect to their work place. The reference group consisted of individuals working in indoor place and having two putative low risk genotypes (positive *GSTM1* and NN genotype of *GSTO2* polymorphism).

**Table 3:** Association between profile of polymorphisms of *GSTO2* (N142D) and *GSTM1*, in relation to work place and cataract risk.

**Work place**	***GSTM1* polymorphism**	***GSTO2* polymorphism**	**Risk factors**	**Control**	**Case**	**OR**	**95%CI**	**p-value**
Indoor	Positive	NN	0	35	21	1.0	Reference	-
Indoor	Positive	ND+DD	1	37	38	1.71	0.84–3.46	0.135
Indoor	Null	NN	1	26	33	2.11	1.00–4.46	0.049
Indoor	Null	ND+DD	2	36	46	2.13	1.06–4.26	0.033
Outdoor	Positive	NN	1	6	7	1.94	0.57–6.56	0.284
Outdoor	Positive	ND+DD	2	5	16	5.33	1.71–16.7	0.004
Outdoor	Null	NN	2	6	12	3.33	1.09–10.2	0.035
Outdoor	Null	ND+DD	3	10	13	2.16	0.81–5.80	0.124

To show the additive effect(s) of *GSTM1* and *GSTO2* polymorphisms and work place on the risk of cataract, the risk associated with the number of putative high risk factors was investigated. Statistical analysis showed that the risk of cataract increased as a function of number of putative high risk factors (Table 4). The results of the present study were criticized because data on the work place, which is known to be associated with risk of cataract, were missed for some control subjects. Using “sensitive analysis” it is possible to estimate the potential effect of this variable on the study by assuming various degree of maldistribution of the variable in the control group and seeing how it would affect the results. As mentioned in “Statistical analysis” section, we test two distributions for the missing data among controls. Statistical analysis showed the same results on association between the number of risk factors and susceptibility to cataract was observed (Table 4). There were significant linear trends for presence of 0, 1 and 2 or more than 2 risk factor and susceptibility to cataract (χ^2^=8.001, p=0.005; under assumption 1: χ^2^=12.10, p=0.001; under assumption 2: χ^2^=6.75, p=0.009). Therefore the main finding of the present study which indicating that outdoor working, null genotype of *GSTM1* and ND+DD genotype of *GSTO2* act additively in relation to susceptibility to cataract. Previously, it has been reported that some polymorphisms have additive effect on risk of other diseases or multifactorial traits [20-24].

**Table 4:** Association between number of risk factors and cataract risk.

**Number of risk factors**	**OR**	**95%CI**	**p-value**
0	1.0	Reference	-
1	1.88	1.00–3.54	0.049
2 and 3	2.54	1.34–4.80	0.004
**Assumption 1**			
0	1.0	Reference	-
1	1.94	1.06–3.54	0.030
2 and 3	2.91	1.58–5.36	0.001
**Assumption 2**			
0	1.0	Reference	-
1	1.79	0.97–3.30	0.062
2 and 3	2.30	1.24–4.25	0.008

Note: In assumptions 1 and 2 all and 50% of missing data for work place were indoor.

In summary, the present study suggested that polymorphisms of *GSTM1* and *GSTO2* and work place are associated additively with increased risk for senile cataract in an Iranian population. Considering that the *GSTM1* and *GSTT1* null genotypes are both associated with increased risk for senile cataract in Asian populations but not in Caucasian populations [12], more epidemiologic studies are necessary to further ascertain the relationship between GST polymorphisms and genetic predisposition to senile cataract.

One of the main limitations of the present study is the measurement of sunlight exposure as a dichotomous variable (indoor versus outdoor). In future, our findings should be confirmed by a large-scale study. It is recommended that in future studies, sunlight exposure is measured as a variable with more categories or as a continuous variable. Difference between cases and controls for age of subjects is another limitation of our study.
